# Everyday Health among Older People: A Comparison between Two Countries with Variant Life Conditions

**DOI:** 10.1155/2017/2720942

**Published:** 2017-08-10

**Authors:** Cecilia Fagerström, Lena Sandin Wranker, Zarina Nahar Kabir, Ola Sternäng

**Affiliations:** ^1^Blekinge Center of Competence, Karlskrona, Sweden; ^2^Department of Health and Caring Sciences, Linnaeus University, 391 82 Kalmar, Sweden; ^3^Department of Health Sciences, Division of Geriatric Medicine, Lund University, Lund, Sweden; ^4^Division of Nursing, NVS, Karolinska Institute, Stockholm, Sweden; ^5^Institute of Gerontology, School of Health and Welfare, Ageing Research Network-Jönköping (ARN-J), Jönköping University, Jönköping, Sweden; ^6^Stockholm Centre for Health and Social Change (SCOHOST), Södertörn University, Huddinge, Sweden

## Abstract

This study described health factors of importance for everyday health, such as pain, tiredness, and sleeping problems, in a cross-national context. Data for persons 60+ years were obtained from the Poverty and Health in Aging study, Bangladesh, and the Swedish National Study on Aging and Care-Blekinge. The strongest associations with everyday health in Sweden were found for pain and tiredness, while in Bangladesh they were financial status, tiredness, and sleeping problems. As similarities were found regarding the associations of tiredness on everyday health, tiredness may be a universal predictor of everyday health in older adults irrespective of country context.

## 1. Introduction

Health and health related quality of life (HRQoL) are concepts that are frequently used in research on older people (persons aged 60 years or more) to increase the understanding of their well-being, although the topics studied and the instruments used vary [1–3]. HRQoL instruments are often limited in their ability to perform contextual comparisons in nations where life circumstances differ, since they are mainly focused on individual's physical and mental health and less on the individual's life situation. An indicator of health that is suitable for most contexts would be valuable and useful. Such an indicator should reflect how older people perceive their current life situation and should be highly affected by their health [4]. Thus, in cross-national studies of high- and low-income nations, a composite variable of health in everyday life might provide an appropriate way of assessing the health situation in advanced age.

Our intention was to develop a short subjective assessment of health and life situation which could be used in countries that vary greatly in economic development. In order to identify common patterns, data were accessed from two population studies, one in Bangladesh and one in Sweden, to compare the results of two contrasting settings in which living conditions are highly different.

Focusing on health and ill health, rather than the diagnoses per se, is rational as older people feel more hindered by their symptoms than by their diseases [5]. Pain and sleeping problems are common symptoms in advanced age. However, although knowledge about pain in the aging population has improved during the last two decades [6, 7], little evidence has been published about how pain and sleeping problems affect an older person's perceptions of current life and whether this relationship differs between countries. Prevalence of sleeping problems and pain vary by disease, clinical setting, gender, age, and country of residence. Pain [8] and impaired sleep [9, 10] are both related to advanced age [8] and general health [8, 10].

Pain is an unpleasant sensory and/or emotional experience [11], but the factors underlying individual differences in pain response are not yet fully understood [12]. The prevalence of pain is however high among older persons, especially women [13, 14]. Normal aging does not necessarily influence perceived health in everyday life in a negative way [15]. The experience of pain is affected by biological, social, or cultural factors [16, 17]. From a contextual perspective, it is interesting to note that pain is more commonly reported and of a greater severity among Bangladeshis than Caucasians living in the United Kingdom [18]. However, the authors of the study did not find evidence of differences between ethnic groups in the impact of pain on perceived health.

Significant variations by ethnicity in sleep characteristics of older men have been observed [19]. Older people appear to have an increased risk of reporting both sleep complaints and low perceived health (low HRQoL), particularly if they live with a high degree of comorbidity [20]. Among older people in rural Bangladesh, the prevalence of multimorbidity is high (53.8%), especially among women [21]. Razzaque and his colleagues [22] investigated the health of older people in Bangladesh and found that individuals who suffered from illness and were in the greatest need of support were older, single women with low education levels and economically disadvantaged. These conditions can also be translated to the Swedish context. It can, therefore, be assumed that pain and insomnia are common conditions in both Bangladesh and Sweden and may be factors that influence everyday health.

Older people, especially in rural areas, are vulnerable in health status and access to health services (Elnitsky and Alexy, 2010). In different settings, the financial status influences the use of healthcare as well as the life situation. In Sweden, income differentials in healthcare-seeking behaviours that favour higher-income groups have been observed among men irrespective of age and among older women [23]. For instance, people with a higher income made almost 50% more healthcare visits than those with lower incomes [23]. Furthermore, perceived illness and financial access to health services are both factors that are known to contribute to accessing treatment in Bangladesh [24]. In summary, people in Bangladesh and Sweden, two countries that differ greatly in living conditions, may have different views of their health in everyday life. The aim of this study was to examine health factors of importance for everyday health in a cross-national context. The two factors comprising everyday health in this study, that is, people's life situation and perceived health, have previously been mentioned as components of self-reported good life in older people [25]. Associations between everyday health and pain, tiredness, and sleeping problems in persons 60 years and older in Bangladesh and Sweden were studied.

## 2. Methods

### 2.1. Sample

Data were collected from two population-based studies. The cross-sectional “Poverty and Health in Aging” (PHA) study was conducted in 2003, and the participants were aged 60 years and above and resided in a rural region, MATLAB, situated approximately 60 km southeast of the capital Dhaka in Bangladesh. The participants were randomly drawn from a demographic register and after they agreed to participate, they first completed a home interview and then had medical and cognitive examinations performed at a nearby health clinic. A total of 625 persons participated in home interviews. For information about dropouts, see Kabir et al. [26]. Approximately 60% of the participants were illiterate. Among the literate, the mean years of education were 4.8, SD 2.8. No one reported that they lived alone and half of the sample (56.7%) was married. Trained interviewers completed the questionnaires based on the responses from the participants.

The baseline sample of the longitudinal Swedish National Study on Aging and Care-Blekinge (SNAC-B) was collected from 2001 to 2003 and comprised 1,402 individuals aged 60 to 96 years. The study population consisted of randomly selected residents living in the Karlskrona municipality, which is situated in southeast Sweden (with approximately 60,600 inhabitants). The participants came from both urban and rural areas and lived both at home and in special housing. The response rate was 61%. Potential participants were sent a letter inviting them to participate in the study. Informed consent was obtained. The examination and testing by research personnel were conducted in two sessions, each lasting about two hours. Data were collected through medical examination, structured interviews, and questionnaires. Those who agreed to participate but were unable to travel to the research center were assessed in their homes. Approximately 70% had primary school education and the rest of the sample had education higher than primary school. Half of the sample was married and lived together with someone (54.5% and 51.6%, resp.). Further information about the protocols of the SNAC-B study can be found elsewhere [27].

In Sweden, 6% of the sample was estimated to have severe cognitive impairment (MMSE scale < 17) and 4% were diagnosed with dementia according to DSM IV in Bangladesh. For participants with cognitive impairment, questionnaires were completed by proxies in order to increase the reliability. Ethical approvals were obtained from the Regional Research Ethics Committees at the Karolinska Institute (Dnr 264/03), the Centre for Population and Health Research at the International Centre for Diarrhoeal Disease Research, Bangladesh (number 2003-025), and Lund University (Dnr LU 128-00, LU 604-00). For characteristics of the participants in the present study, see Table 1.

### 2.2. Measures

The variables were reviewed to ensure that the questions from the two databases (PHA and SNAC-B) were the same. Two other criteria taken into consideration were that the response options were adjusted to be equal and that the correlations between everyday health and the main variables were similar on an overall level in the two countries. The everyday health variable was constructed as the sum of the responses to two questions: “(A) How is your life situation?” with the response options of very poor (1), poor (2), rather good (3), or very good (4) and “(B) Compared to other people of your age, how do you feel your health is?” with not as good as others (1), as good as others (2), or better than others (3), as the possible responses.

The everyday health variable had six categories (2–7) ranging from a very poorly rated life situation and health (2) to a very highly rated life situation and health (7). The correlation between the two questions (A, B) was 0.33 (*p* < 0.001) in the PHA study and 0.19 (*p* < 0.001) in the SNAC-B study.

#### 2.2.1. Age and Gender

Age and gender were included as demographic variables. In Bangladesh, birth certificates did not exist for all in the age group in the study; in such cases, age was determined based on relevant biological and historical events in the person's life. This method of determining age in low-income countries is well established [28, 29].

#### 2.2.2. Worried about Money

This variable was based on the question “Are you worried about having enough money for the household?” with the alternatives yes (1) and no (2).

#### 2.2.3. Have Cash

This variable was related to the participant's financial situation and was based on the question “Do you always have some cash?” The response options for this variable were no (1) and yes (2).

#### 2.2.4. Problem in Bending

This was assessed with the question “Do you have any problem bending forward?” with the two alternatives yes (1) and no (2).

#### 2.2.5. Pain

The question “Do you suffer from bodily pain?” was asked with three options: yes (1), sometimes (2), and no (3).

#### 2.2.6. Feel Tired

Tiredness was based on the question “Do you usually feel tired?” with the response alternatives yes (1) and no (2).

#### 2.2.7. Problem Sleeping

The question “Do you have trouble sleeping?” was used. This question had two response options: yes (1) and no (2).

### 2.3. Statistical Analysis

The analyses were conducted in SPSS version 22 (SPSS Inc., Chicago, IL, USA). Alpha levels were set at 0.05. Age, gender and financial situation, tiredness, problem sleeping, pain, and the outcome variable of everyday health were presented with their descriptive statistics for each country (see Table 1). Spearman's rho (*r*_*s*_) was used to calculate the correlations between the independent variables and everyday health. A hierarchical regression model was performed on the total sample (see Table 2). In the first step, the background variables of age, gender, worrying about money, and having no cash were entered into the model as control. In the second step, the potential predictors of problem bending, pain, feeling tired, and problem sleeping were included. In the third step, the country variable was entered, and finally, interactions between country and each of the independent variables were entered. Everyday health was the outcome variable. Regression models were also performed separately for the two countries.

## 3. Results

The total sample included 2,027 participants; 1,402 (69%) were drawn from the Swedish study SNAC-B. Participants from Bangladesh were younger, had less financial resources, felt more frequently tired, and experienced pain and mobility problems more often (*p* values < 0.001). Approximately 70% of the sample in Bangladesh stated that that they did not have enough money. The corresponding proportion in Sweden was 6%.

**Figure 1:**
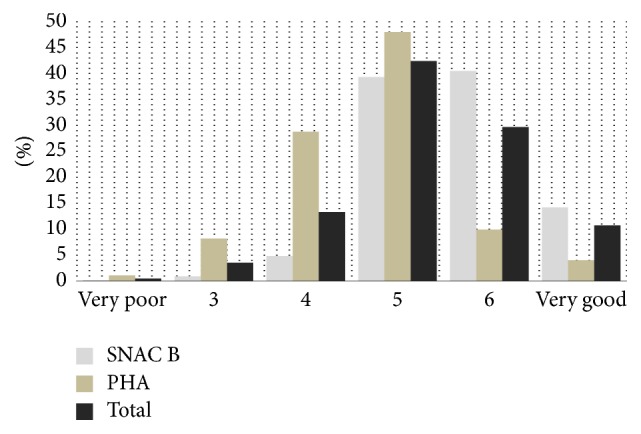
The distribution of the everyday health variable. Note: the everyday health variable was constructed as the sum of two questions: “How is your life situation?” (with the response alternatives very poor to very good (1–4)) and “Compared to other people your age, how do you feel your health is?” (with not as good as others, as good as others, or better than others as the possible responses (1–3)). The resulting variable “everyday health” had six categories (2–7) ranging from a very poorly rated life situation and health (2; very poor) to a very highly rated life situation and health (7; very good). PHA = Poverty and Health in Aging in Bangladesh, SNAC-B = Swedish National Study on Aging and Care-Blekinge.

The distribution of the everyday health variable is presented in Figure 1. Approximately 40% of the sample reported that they had good everyday health (score 5), and one-tenth rated their everyday health as very good (score 7). Higher percentages of older persons in Sweden reported better everyday health compared to their counterparts in Bangladesh. In the total sample, the relationships between everyday health and life situation, everyday health and health compared to others, and life situation and health compared to others were *r*_*s*_ = .875, *r*_*s*_ = .740, and *r*_*s*_ = .315, respectively. When the country samples were separated, the significant relationships between the variables remained, but the relationships were found to be slightly weaker in the Swedish sample.

### 3.1. Associations of Everyday Health with Pain and Sleeping Problems in the Total Sample

A hierarchical model was first performed for the total sample to examine the predictors of everyday health. After controlling for age, gender, and financial situation, the variables entered in step 2 (problem bending, pain, feeling tired, and problem sleeping) increased the explained variance (*R*^2^) in everyday health by .08 (from .23 to .31) (see Table 2). Entered variables in steps one and two were significant. When the country of residence was included in the model (step 3), *R*^2^ increased only slightly. In the last step (step 4), the eight two-way interactions between country of residence and each independent variable increased the explained variance by .03 to a total *R*^2^ = .35. Two significant interaction effects were detected: pain × country (*β* = −.149) and having no cash × country (*β* = −.102). Age, gender, worrying about money, pain, feeling tired, problems sleeping, and country of residence remained significant in the final model.

### 3.2. Associations of Everyday Health with Pain and Sleeping Problems by Country of Residence

The data was also analysed through a hierarchical regression model for each country to establish whether there were any country-specific predictors of everyday health. This model consisted of two steps (see Table 3). In the first step, gender was significant in SNAC-B (*β* = −0.071), and gender (*β* = −0.123), worrying about money (*β* = −0.221), and having no cash (*β* = −0.227) were significant in PHA. The second step, in which the predictive variables were included, increased the explained variance (*R*^2^) in everyday health by .11 for participants in SNAC-B and by .07 for participants in PHA. After controlling for background variables in SNAC-B, pain (*β* = −.306) and feeling tired (*β* = −.076) were significant predictors of everyday health. Corresponding figures in PHA were feeling tired (*β* = −.155) and problem sleeping (*β* = −.127).

## 4. Discussion

A composite variable of health and life situation that generates a subjective assessment of everyday health may provide us with new knowledge. This study of older people is the first to include the concept of everyday health. We assessed everyday health of older people's perceptions of their health compared with peers in a high- and in a low-income country. The purpose of the study was to examine the associations between everyday health and potential predictor variables such as pain, tiredness, and sleeping problems as well as any country-specific association between these variables. Low perceived everyday health was reported most commonly by women. The results indicated that older people's perceptions of everyday health can be explained by pain, sleeping problems, and the sociodemographic variables assessed in this study. Health problems and sociodemographic variables explained 35% of the variance in everyday health, but country-specific patterns still existed.

The concept of everyday health is focused on a person's present situation and reflects how older people perceive their current health and life. The present study confirmed that health issues are central but do not equate to people's perception of their life situation. This distinction justifies our choice to combine the two measures of HRQoL into a composite variable.

The differences between the two countries in this study were that pain and tiredness had the greatest impact on everyday health in Sweden, while financial status, tiredness, and sleeping problems had the highest impact on everyday health of older people in Bangladesh. A notable finding was that pain was associated with everyday health in Sweden only and was the main factor affecting their everyday health. Those with pain had a considerably higher probability of impaired everyday health compared to their counterparts with no pain. This pattern is consistent with a recent study of the same sample from SNAC-B, in which pain was found to be strongly associated with low HRQoL [30]. However, in that study, this relationship was significant only in women. Similarly, Hawkins and colleagues [31] found that pain was strongly associated with low HRQoL among older adults after controlling for demographic, socioeconomic, and health status characteristics. In the present study, pain was not a predictor of everyday health in Bangladesh despite the fact that pain was reported more often than in Sweden. One possible explanation could be that financial survival in a low-income country such as Bangladesh may be overriding physical problems, whereas in a high-income country such as Sweden where financial worries are not predominant, the focus is on physical discomfort. It is notable that pain treatment has a low priority among Bangladeshis [cf. [32]], whereas people in Sweden may assume that pain treatment, or at least pain relief, is included in the standard treatment.

As similarities between the two countries were found regarding the effects of tiredness on perceptions of everyday health, tiredness may be considered as a universal factor of everyday health irrespective of country context. However, significant between-country differences were also found. Having sleeping problems influenced a person's everyday health only in Bangladesh, and feeling tired influenced everyday health in both countries. Impaired sleep was commonly reported in both countries, which is supported by other studies on older adults [9, 10]. In old age, sleep is often experienced as normal by an older person even if the sleep is impaired. For instance, it has been stressed that while their sleeping patterns could be irregular [33], older people tend to adapt to age to variations in their sleeping patterns. However, as sleeping problems are associated with everyday health and are commonly reported in the population, irrespective of country, this needs to be considered further.

In the analyses, country-specific factors on everyday health were identified. The differences in results may be dependent on the sociocultural factors in the investigated country. For example, financial status may universally influence the perceptions of everyday health, but its meaning differs between high- and low-income contexts. Financial situation significantly affected older people's everyday health in Bangladesh but not in the Swedish population. Also, Diener [34] stressed that the factors influencing people's perception of HRQoL may vary in different societies. However, these results require further attention as the majority of the population in Bangladesh reported a poor financial situation compared with approximately only 6% of the population in Sweden. It could be assumed that this difference between the two countries depends on the infrastructure of the societies, for example, each country's pension system, which may in turn influence the residents' perceptions of their current life situation more than their health conditions. The differences may also be explained by other sociodemographic characteristics, such as marital status, income, and leisure activities, which have a significant impact on older people's life situations [22, 35]. Finally, in-country geographical area may also play a role in the proportion of people in poor financial situations, as the sample in Bangladesh lived in a rural area, whereas the Swedish sample lived in both urban and rural areas.

While sleeping problems and pain [36] as well as economic situation [37] were expected to be closely associated with each other, it is still not clear whether these findings indicate that the symptoms are equally prominent in different countries. Having a low perceived everyday health was most commonly reported among women. As older women in general have fewer material resources than men [38], it can be assumed that it is not being a woman that influences everyday health but rather the financial situations of women compared with men. In future studies, it would be useful to investigate health problems with a particular focus on different types of pain and sleep problems and, if possible, how poor financial situations influence everyday health among older persons.

The collection of data in different contexts increases the risk of bias [39], but the benefits may exceed the disadvantages. For example, the same question might not necessarily have the same meaning in different cultures [40]. The outcome variable everyday health was based on three criteria (see Methods) to minimize this cultural difference. The benefit is the validation of results when data are collected in a similar way in different contexts and a similar phenomenon is described; this increases the opportunity to generalise results [41]. More information related to the participants' everyday health, for instance, sociodemographic factors, mood and depression symptoms, morbidity pattern, and healthcare utilization, may be important to include to verify subjective health information and to describe country-wise differences. Not including such additional health data can be seen as a limitation of the study, but no such comparative data were available in the two data bases. Although much of this type of information exists in the two databases, these variables were measured differently in the two countries. Since the focus of the present study was on comparisons between the two samples in Sweden and Bangladesh, we refrained from including these variables in the analyses. Furthermore, to control for unique and country-specific variations in background variables, the final models were constructed separately for each country in addition to the combined model. Anyhow, the results must be carefully interpreted. Further analyses are of interest to gain a deeper understanding of the relationship between everyday health and the commonly used HRQoL as well as QoL.

## 5. Conclusions

We suggest that the everyday health variable can be applied in countries that vary greatly in economic development as a subjective assessment of a person's health and life conditions. There were similarities between the countries regarding the factors associated with everyday health. The difference between the two countries was that pain and tiredness had the greatest impact on everyday health in Sweden, whilst financial status, tiredness, and sleeping problems had the highest impact in Bangladesh. The results of the study can help community health providers and administrators strategically plan to meet older people with health problems at risk for decreased everyday health and to meet their healthcare needs in different country contexts.

## Figures and Tables

**Table 1:** Description of the participants from the PHA (*n* = 625) and the SNAC-B (*n* = 1402) studies.

Variables	PHA	%	SNAC-B	%	*p* value
Age mean (SD)	69.6 (7.1)		76.7 (10.2)		<0.001
Women (*n*)	345	55	795	58	n.s.
Worried about money (*n*)	447	72	77	6	<0.001
Have not cash (*n*)	383	61	232	19	<0.001

Problems bending (*n*)	234	37	128	10	<0.001
Sometimes have pain (*n*)	246	39	800	66	
Often or always have pain (*n*)	252	40	69	6	<0.001
Feel tired (*n*)	196	31	158	13	<0.001
Problems sleeping (*n*)	175	28	376	30	n.s.

Life situation mean (SD)	2.9 (0.5)		3.2 (0.5)		<0.001
Health compared to others mean (SD)	1.8 (0.6)		2.4 (0.6)		<0.001
Everyday health mean (SD)	4.7 (0.9)		5.6 (0.8)		<0.001

*Note*. Differences between the two samples were tested for significance with *t*-tests (for means) or Chi square tests (for percentages). PHA = Poverty and Health in Aging in Bangladesh. SNAC-B = Swedish National Study on Aging and Care-Blekinge.

**Table 2:** Everyday health and its associations with the background and predictor variables in the total sample.

	*β*	*p* value	Cumulated *R*^2^
Step 1			0.23
Age	.066	0.003	
Gender (reference: women)	−.078	<0.001	
Worried about money	−.349	<0.001	
Having no cash	−.162	<0.001	
Step 2			0.31
Problems bending	−.063	0.012	
Pain	−.198	<0.001	
Feeling tired	−.111	<0.001	
Problems sleeping	−.069	0.001	
Step 3			0.32
Country	.208	<0.001	
Step 4			0.35
Age × country	.008	0.711	
Gender × country	−.029	0.175	
Worried about money × country	−.048	0.159	
Having no cash × country	−.102	<0.001	
Problems bending × country	−.009	0.759	
Pain × country	−.149	<0.001	
Feeling tired × country	−.022	0.390	
Problems sleeping × country	−.037	0.083	

**Table 3:** Everyday health and its association with the background and predictor variables in the two samples from Sweden and Bangladesh.

	SNAC-B (*n* = 1,402)			PHA (*n* = 625)		
	*β*	*p* value	Cumulated *R*^2^	*β*	*p* value	Cumulated *R*^2^
Step 1			.01			.15
Age	.052	0.103		.017	0.651	
Gender (women reference)	−.071	0.026		−.123	0.002	
Worried about money	−.056	0.089		−.221	<0.001	
Having no cash	−.012	0.709		−.227	<0.001	
Step 2			.12			.22
Problems bending	−.032	0.302		−.080	0.068	
Pain	−.306	<0.001		−.047	0.268	
Feeling tired	−.076	0.014		−.155	<0.001	
Problems sleeping	−.054	0.086		−.127	0.001	

*Note*. Hierarchical regression on data from the two databases SNAC and PHA. PHA = Poverty and Health in Aging in Bangladesh; SNAC-B = Swedish National Study on Aging and Care-Blekinge.
